# Green and rapid preparation of long-term stable aqueous dispersions of fullerenes and endohedral fullerenes: The pros and cons of an ultrasonic probe

**DOI:** 10.1016/j.ultsonch.2021.105533

**Published:** 2021-03-19

**Authors:** Ivan V. Mikheev, Mariya O. Pirogova, Liliia O. Usoltseva, Anna S. Uzhel, Timofey A. Bolotnik, Ivan E. Kareev, Viacheslav P. Bubnov, Natalia S. Lukonina, Dmitry S. Volkov, Alexey A. Goryunkov, Mikhail V. Korobov, Mikhail A. Proskurnin

**Affiliations:** a Chemistry Department Analytical Chemistry Division of Lomonosov Moscow State University, 119991 Moscow, Russia; b Chemistry Department Physical Chemistry Division of Lomonosov Moscow State University, 119991 Moscow, Russia; c Institute of Problems of Chemical Physics of the Russian Academy of Sciences, 142432 Chernogolovka, Moscow Region, Russia

**Keywords:** Fullerene, Endofullerene, Aqueous fullerene dispersion, Ultrasound-assisted preparation, Immersible ultrasound probe, Sonochemistry

## Abstract

A green, scalable, and sustainable approach to prepare aqueous fullerene dispersions (AFD) C_60_, C_70_, endohedral metallofullerene Gd@C_82_, and their derivatives C_60_Cl_6_, C_70_Cl_10_, and supramolecular and ester-like derivatives, 10 fullerene species total, is proposed. For the first time, an immersed ultrasonic probe was used to preparing dispersions for pristine fullerenes without addends. Both ultrasound-assisted solvent-exchange and direct sonication techniques for AFD preparation using an immersed probe were tested. The average time for AFD preparation decreases 10–15 times compared to an ultrasound-bath-assisted technique, while final fullerene concentrations in AFDs remained at tens of ppm (up to 80 ppm). The aqueous dispersions showed long-term stability, a negatively charged surface with a zeta potential up to –32 mV with an average nanocluster diameter of no more than 180 nm. The total anionic and cationic compositions of samples were found by inductively coupled plasma atomic emission spectroscopy and chromatographic techniques. The highlights and challenges of using an ultrasound probe for AFD production are discussed.

## Highlights

• Rapid ultrasound-probe preparation of aqueous fullerene dispersions (AFDs).
• Sonication probe is more effective for AFD preparation than a bath.
• AFDs have up to 80 ppm concentrations, 180 nm mean diameter, –32 mV *ζ*-potential.
• AFDs of C_60_, C_70_, Gd@C_82_, etc. showed long-term storage and aggregate stability.
• Complete characterization and metal/organic impurities control of AFDs is proposed.

## Graphical abstract

**Figure:**
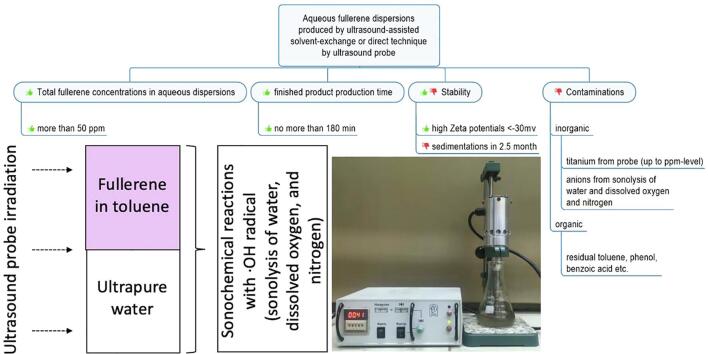

## 1 Introduction

Aqueous dispersions of fullerenes (AFD) are relevant in various applications and research. Unique physical and chemical properties of fullerenes make them promising for medical and materials-science claims; however, much work on AFDs deals with hydrophilic functionalization, which is often necessary [1] in photodynamic therapy [2] and magnetic resonance imaging (MRI) [3] for endohedral fullerenes (endofullerenes); their biomedical and others applications are reviewed in [4]. However, in many instances, such functionalization of fullerenes should be avoided, whereas an AFD material is still required. Thus, due to ultralow water solubilities of nonfunctionalized fullerenes [5], [6] it is compulsory to apply alternative approaches for producing their AFDs — by solvent exchange [7] dialysis [8] ultrasonication [9] complexation [10] etc.

To date, there are several ways to produce stable, highly concentrated, and small-sized distributed AFDs: the pioneering work in this field, an ultrasound-assisted solvent-exchange technique for C_60_ and C_70_ [11], stable dispersions for C_60_ and C_70_ [12] (first mentioning of the purification by nitrogen purging), a novel dialysis technique for first large-scale synthesis [13] and approaches to bioconjugate materials with monomolecular solutions [14], [15]. It is noteworthy that stable aqueous dispersions of nonfunctionalized species are topical not for fullerenes only but other carbon allotropes [5], [16].

Ultrasonic technologies are beginning to take priority in chemistry, especially green chemistry. Many applications in materials science, organic synthesis, analytical chemistry, etc. have been developed [17], [18], [19]. The use of ultrasound sources as probes or sonoreactors in extraction helps to increase recoveries [20]. The energy of ultrasound causes cavitation in solvents, which generates numerous tiny bubbles, creates pressure and temperature to digest and form homogenized colloidal or other samples [21]. Concerning this, ultrasound solubilization is the most frequently used approach to produce carbon-allotrope aqueous dispersions [22], [23]. Two types of ultrasonic sources, baths and immersible probe-based sonicators, have been well compared in terms of reproducibility, price, and frequency of use [24]. The use of ultrasonic probes instead of ultrasound baths for producing colloid systems of carbon nanomaterials is gaining broad momentum over the past five years [25] and were used to prepare fullerene derivatives and dispersions [25], [26], [27].

However, none of the studies on ultrasound-assisted preparation of AFDs show sufficient characterization of resulting samples from the viewpoint of impurity composition. To be usable, AFDs not only have to be highly concentrated but also free of impurities of both organic and inorganic nature — the most sensitive to these parameters are antioxidants and MRI contrast agents. Still, there is little research on the decomposition of ultrasonic probes in action. However, the resulting samples may contain sufficiently large amounts of their material (usually titanium), and its species has not been proven [28]. Also, ultrasound facilitates the breakdown of carbon–carbon and other chemical bonds that may affect the AFD composition [24], [29]. Firstly, there are various unsolved problems concerning sonochemical formation of byproducts (products of oxidation of main components) and the reproducibility of the composition [30]. Secondly, the residual amounts of organic solvents can be decomposed or at least oxidized by ultrasound [31]. Finally, fullerenes are oxidizing agents, in particular when exposed to UV/vis radiation, and they thus may yield reactive oxygen species [32], [33]. The triplet states of C_60_ and C_70_ transfer energy efficiently to ^3^O_2_ giving singlet molecular oxygen (^1^O_2_) [32] but it is investigated for organic solutions for C_60_ [34] and C_70_ [35] only. Nevertheless, their antioxidant activity for non-functionalized or slightly functionalized fullerene species should be investigated. Thus, a complete understanding of the capabilities of fullerene aqueous dispersions, especially for biomedical applications, is required for both the ultrasonic-assisted preparation and full characterization of the resulting AFD samples.

Hence, this study aims (*i*) to compare the preparation of aqueous fullerene dispersions using an immersed ultrasound probe with ultrasound-bath-assisted preparation and (*ii*) to characterize the main product and impurity composition of the prepared materials. Also, we report on pitfalls related to preparing aqueous dispersions as well as their characterization. In our opinion, this will make it possible to prepare reference materials for a bioanalytical evaluation of the safety and potential toxicity of aqueous dispersions of the fullerene family.

## 2 Material and methods

### 2.1 Techniques

#### 2.1.1 Chromatographic and spectroscopic equipment

An Agilent 720 ICP-OES spectrometer (Agilent, Australia) with an axial view was used for elemental analysis (Ag, Al, As, B, Ba, Be, Cd, Co, Cr, Cu, Gd, Fe, Li, Mn, Mo, Ni, Pb, Sb, Se, Si, Sn, Sr, Ti, Tl, V, W, Zn, Zr, Ca, Mg, K, Na, S, and P). The ICP-OES operating conditions are given in Supplementary Data (Section S.1). An Agilent Cary 4000 spectrophotometer was used for UV/Vis spectra recording. An ISC-1600 chromatograph (Thermo Fisher Scientific, UK) for the separation of anions (${F^{-},{Cl}^{-},NO}_{3}^{-},{SO}_{4}^{2-},{PO}_{4}^{3-}$) equipped with a particular chromatographic column Dionex IonPac AS4 (4 mm I.D. × 250 mm) with a precolumn Dionex IonPac AG4 (4 mm I.D. × 50 mm) was used. For the determination of volatile organic compounds (VOCs), a headspace gas chromatography (HS-GC–MS) was used: a GCMS-QP2010 Ultra Gas Chromatograph Mass Spectrometer (Column: Agilent CP-5 Sil 30 m × 0.25 mm × 0.4 μm) from Shimadzu Europa GmbH, Germany. A HPLC Agilent 1200 with UV–Vis and fluorescence detectors (Diode-Array Detection, a column for phenol determination: Synergi Hydro-RP (Phenomenex, USA) 250 mm × 4.6 mm × 4 μm; a column for benzoic acid: Eclipse XDB-C18 (Agilent, USA) 150 mm × 4.6 mm × 5 μm, and precolumn Security Guard C18 (4 × 3 mm; Phenomenex, USA)) was used. Chromatography operating conditions and chromatograms are given in Supplementary Data (Sections S.2 and S.3). All operating conditions for all instruments are indicated in supplementary data for a detailed description.

#### 2.1.2 Other instruments

The procedures of preparing AFDs by direct or solvent-exchange methods were used for a commercially available off-the-shelf ultrasound probe with a timer MEF93.T (LLC MELFIZ-ul'trazvuk, Russia). The ultrasound probe has an operating frequency 22.00 ± 1.65 kHz, which works in a continuous mode of exposure to ultrasonic energy. In this work, two different ultrasonic tips (surface areas 0.63 ± 0.02 and 6.61 ± 0.02 cm^2^), which provided intensity range (up to 250 W/cm^2^) in two electrical-power modes (0.3 and 0.6 kW) were used. Ultrasound tips were made of titanium alloys, grade TM3 (ISO 28401:2010). Parameters of the ultrasonic device and the operating modes are given in Supplementary Data (Section S.5).

The colloidal parameters of aqueous fullerene dispersions (particle size distribution and zeta-potential) were found by dynamic light scattering (DLS) using a ZetaSizer Nano ZS (Malvern Instruments, UK) operating at 25 °C, the angle of backscattering, 173°.

MALDI mass spectra (positive and negative ion modes) were acquired using a Bruker AutoFlex II reflector time-of-flight mass spectrometer equipped with an N_2_ laser (337 nm, 2.5 ns pulse). *Trans*-2-[3-(4-*tert*-butylphenyl)-2-methyl-2-propenylidene]malononitrile (DCTB, 98%, Sigma–Aldrich) was used as a matrix, the matrix-to-analyte molar ratio in spotted probes being above 1000:1. The samples were prepared using a so-called “sandwich” method. This technique involves the AFD analyte deposition as an aqueous solution followed by water evaporation. Then, a thin layer of DCTB matrix is droped as a toluene solution followed by solvent evaporation.

To estimate the accuracy of UV/vis measurements, total organic carbon analysis (TOC-analysis) has been carried out. The TOC-II (Elementar, Germany) was calibrated by KHP (Potassium hydrogen phthalate) (Merck, Germany) standard solution in a range from 0.1 to 500 ppm.

### 2.2 Reagents, solvents, and standard reference materials

Pristine C_60_ and C_70_ (99+% HPLC-grade) were purchased from NeoTechProduct LLC (Russia) and used as starting materials for further synthesis of other fullerenes derivatives in this work.

The soot containing the Gd@C_2_*_n_* EMFs (total content of Gd atoms up to 4 wt% checked by ICP-OES, and the value of total Gd was recalculated to the general formula of the molecule, Gd@C_82_) has been synthesized by the evaporation of the composite graphite electrodes compounded by gadolinium in the electric arc reactor as previously described [36].

Fullerene C_60_ and C_70_ derivatives were synthesized according to well-known methods [37], [38]; synthetic details are given in Supplementary Data (Section S.7). Phenyl-C_61_-butyrate methyl ester (PCBM) powder was used from Nano-C, 99%. HPLC grade C_60_Cl_6_, C_70_Cl_10_, pyrollidinofullerene bearing two pyridyl groups (Py-C_60_), and C_60_-Pyrollidin-BHT (*N*-methyl-2-[3,5-di-*tert*-butyl-4-hydroxyphenyl]pyrrolidine fullerene derivatives) were used.

ISO grade reagents (nitric acid, phosphoric acid, and acetonitrile) from Panreac, Spain were used throughout. Sodium carbonate, sodium bicarbonate, and benzoic acid (all from Merck, Germany) were used to manage chromatographical measurements. Standard reference materials (SRM) and quality control standards of required elements with certified values (Inorganic Ventures^TM^, USA) were used to conduct ICP-OES measurements. SRMs of phenolic and volatile organic compounds (LLC Ecros-Analytica, Russia) were used. A 20 ppm (in 5 wt% HNO_3_) scandium solution was used as an internal standard. Ultrapure water Milli-Q® Type (Merck, Germany) was applied during the research (TOC < 3 ppb). Neat solvent benzene, toluene, and *o*-xylene of analytical grade as purchased (Component Reagent LLC, Russia) were used to dissolve fullerenes and endofullerenes, respectively. Strata-X Polymeric solid-phase extraction (SPE) cartridges were used for the purification of hydrophobic analytes.

### 2.3 Sample preparation procedures

For all AFD synthesis techniques, an ultrasound probe with a large area of ultrasound horn tips was used (see Supplementary Data, Sections S.4 and S.5). The electrical power was 0.6 kW. In all experiments, ultrapure water of Milli-Q® Type was used. AFD obtained using direct (DM) or solvent-exchanged ultrasound-assisted (SE-US or SE-probe) procedures.

**Procedure 1. Synthesis of aqueous fullerene and endofullerene dispersions by the ultrasound-assisted solvent-exchange process.** A weighed (*ca.* 0.0150 mg) portion of fullerenes is placed into a 250-ml conical flask. Then, 10 ml of neat toluene is added. The solution is exposed to ultrasonic treatment for 1 min. Then, 150 ml of ultrapure water is added. The two-phase solution is exposed to ultrasonic treatment for 30 min 4 times intermittently with 1 h intervals until the complete evaporation of toluene and the formation of the aqueous fullerene dispersion (a saturated colored solution, which color depends on the fullerene type). Next, the prepared solution is boiled for 15 min (*ca.* 100℃), filtered through a 0.45 μm filter and diluted to 150 ml by ultrapure water. After checking residual organic compounds content by HS-GC–MS, the solution was purifed, if necessary, by solid-phase extraction (SPE) cartriges with a commercially available polystyrene-divinylbenzene copolymer (PS/DVB) resin.

**Procedure 2. Synthesis of aqueous fullerene and endofullerene dispersions by ultrasonication of immiscible water and solid fullerene without organic solvents.** Weighed (*ca.* 0.0150 mg) portion of fullerenes is placed into a 250-ml conical flask. Then, 150 ml of ultrapure water is added. The solution is exposed to ultrasonic treatment for 30 min 4 times intermittently with 1 h intervals and then is diluted to the 150 ml by ultrapure water until the the formation of aqueous fullerene dispersion (a saturated colored solution, which color depends on the fullerene type). Next, the prepared solution is filtered through a 0.45 μm filter.

**Procedure 3. Sonication of ultrapure water. Ultrasound probe decomposition experiment.** 150 ml of ultrapure water is added to a conical flask. This sample is exposed to ultrasonic treatment for 120 min several times. The time of exposure is 1, 5, 10, 30, 60, and 120 min. For treatments during 60 and 120 min, sonication is conducted every 30 min intermittently with 1 h intervals. Then, the sample is diluted to the mark with 150 ml of ultrapure water.

**Procedure 4. Sonication of ultrapure water with neat toluene (or benzene).** A mixture of 150 ml of ultrapure water and 10 ml of neat toluene (or benzene) is added to a conical flask. The solution is exposed to ultrasonic treatment for 120 min (4 times every 30 min with 1 h intervals) until toluene (benzene) evaporation and then is diluted to the mark with 150 ml of ultrapure water.

## 3 Results and discussion

The choice of fullerenes in this study is defined by their chemical properties and target use. First, they are the most investigated and non-functionalized C_60_ and C_70_. As for Gd@C_82_, this is a promising MRI contrast agent [4]. For several issues of targeted drug delivery, it is necessary to obtain dispersions of derivative fullerenes [39]; here, we used easily accessible chlorofullerenes C_60_Cl_6_ and C_70_Cl_10_, which are key precursors for the synthesis of functional fullerene derivatives [40]. As well, such derivatives as supramolecular associates of pyrollidinofullerene [1] or ester-like PCBM fullerene have several essential properties for biomedical applications due to their antioxidant activity [41] and electron transfer [42] (see additional information and structure of used samples in Supplementary Data, Section S.6).

### 3.1 Preparation of aqueous fullerene dispersions and their parameters

Fullerenes are difficult to disperse into an aqueous solution owing to strong van der Waals attractive forces and huge hydrophobic interactions between non-polar fullerene molecules. Dispersions obtained by ultrasound-assisted techniques showed superlative negative zeta-potential values and colloidal stability. Sample preparation was conducted according to procedures 1–4. To date, the ultrasound-assisted solvent-exchange procedure for AFD production has been worked out for ultrasonic bath devices. The optimum fullerene concentrations, ratio of the aqueous and organic phase with dissolved fullerene, and the time of ultrasonic treatment have already been determined for C_60_, C_70_ [43], [44] and Y@C_82_ [45]. In this work, we projected our previously obtained data on the ultrasound-bath-assisted solvent-exchange process [43], [44], [45], [46] onto an immersed ultrasound probe. On the other hand, an ultrasound probe was previously used for the preparation of fullerene derivatives only [25], [47]. Due to more affordable ultrasound equipment, it was necessary to find the appropriate operation time.

We found that after 30 min of ultrasonic treatment, the flask and aqueous medium strongly heat up to 55–65℃; in the case of water/toluene treatment, overheating is much higher, up to 70–85℃. Similar behavior of the system, heating up to 85℃ during the sonication, was observed previously [48]. Therefore, the optimum procedure for preparing the dispersion is to repeat the process several times for 30 min with interruptions of 15 min to cool down.

It is proven in [49] that temperature significantly affects the aggregation of carbon nanotubes. Obviously, at lower temperatures, we have: (*i*) a reduced level of Van der Waals energy between fullerenes molecules, (*ii*) a reduced frequency of collisions and contacts between fullerenes because Brownian motion is temperature-dependent. In general, this explains why the yield increases for the ultrasonic probe (with higher temperature) compared to the ultrasonic bath (see Table 3).

If the preparation process is continuous, we (*i*) stop at the full evaporation of the aqueous or organic phase before AFD is fully produced or (*ii*) required yields (*ca.* 100%) of the target product are not reached. However, using a probe, we have obtained a much better yield in comparison with a bath sonicator for pristine C_60_ and C_70_ (up to 10–15%). Empirically, the concentration of fullerenes and ultrasound energy supplied showed perfect correlation. The concentration of C_60_ of ~ 68 ppm was obtained by bath sonication for 120 h [43] while the same yield was achieved for 5 h using a probe sonicator (Table 1, Table 3), i.e. 24 times faster than using a bath. Thus, decreasing the time of sonication at the same level of fullerene yield is caused by the quantity of acoustic energy delivered to the suspension. The exact correlation was shown for graphene suspensions [50].

Looking critically at the data (Table 3), one can conclude that using a ultrasound bath in SE mode gives the best results. On the contrary, for an ultrasound probe, both DM and SE techniques most likely get the same yield. (i) The high vapor pressures of non-aqueous solvents render them incapable of sustaining cavitationally induced reactions [51]. For the used solvents, the temperature at which vapor pressure equals 100 kPa [52] is 110.1^◦^C for toluene and 99.6^◦^C for water. (ii) The longitudinal velocity of ultrasound at 20^◦^C is 1360 m/s for toluene, and 1480 m/s for water [53] which may reduce efficiency of proposed SE-US probe techniques.

### 3.2 Fullerene concentration in AFDs

All analytical and stability parameters of prepared dispersions are presented in Table 1. Even though the ultrasound probe is *ca.* 10 times much more potent and effective in comparison with an ultrasonic bath, the previously reported concentrations for an ultrasonic bath of 150 ppm for C_60_ were not reached [43]. Although the average time for preparing dispersions using an ultrasound probe decreases 10–15-fold. In all the cases—both pristine fullerenes C_60_ and C_70_ and fullerene targeted derivatives C_60_Cl_6_, C_70_Cl_10_, Py-C_60_, PCBM, and C_60_-Pyrollidin-BHT— we observed the same anionic composition of the samples at a ppm level and a comparable appearance of titanium in the samples. A 5- to 7-fold growth of chloride content in AFD of chlorofullerenes in comparison with those for bare fullerenes evidences a partial degradation of the chlorofullerenes during ultrasonic treatment. As for the organic components, no significant amounts were found due to organic-solvent-free synthesis. As well, the upper limit for prepared concentrations in AFD of fullerene derivatives was estimated by TOC-analysis as 20 ppm. The total concentrations of C_60_ and C_70_ were estimated by apparent molar absorptivities [43] at the absorbance maximum using UV/vis spectra. Baseline spectra correction was made using ultrapure water.

**Table 1:** Sonication time, total fullerene concentrations, and organic (residual solvents and their derivatives) and inorganic (cations and anions) compositions of aqueous fullerene dispersions. *n* = 3, *P* = 0.95. DM-probe is a synthesis of aqueous dispersions by direct sonication; SE-US-Probe is a synthesis of aqueous dispersions by ultrasound-assisted solvent exchange procedure. In all cases, the quantities of ${SO}_{4}^{2-}$ and ${PO}_{4}^{3-}$ were less 0.5 ppm; for benzene and ethylbenzene, the sum of *m*- and *p*-xylene, styrene, and *o*-xylene was<0.001 ppm.

Aqueous fullerene dispersion (AFD)	Type of AFD	Time of sonication (running for), h	average concentration of fullerenes by UV/Vis and TOC- analysis ppm	Ti, ppm	F^–^, ppm	Cl^–^, ppm	${NO}_{3}^{-}$, ppm	Phenol, ppm	Toluene, ppm	Benzoic acid, ppm	Size, nm	Zeta potential, mV
C_60_	DM-Probe	1 h 5 times	68 ± 2	0.6 ± 0.1	<0.3	0.8 ± 0.3	2.7 ± 0.3	<0.002	<0.001	<0.005	125 ± 7	–22.9 ± 0.2
C_70_	DM-Probe	1 h 5 times	62 ± 3	1.0 ± 0.1	<0.3	0.7 ± 0.2	3.4 ± 0.3	<0.002	<0.001	<0.005	160 ± 10	–19.0 ± 0.4
C_60_	SE-US-Probe	1 h 5 times	72 ± 4	0.60 ± 0.1	<0.3	3.3 ± 0.3	0.8 ± 0.2	0.035 ± 0.002	1.0 ± 0.2	0.17 ± 0.01	130 ± 5	–21.0 ± 0.5
C_70_	SE-US-Probe	1 h 5 times	68 ± 3	0.5 ± 0.1	<0.3	1.5 ± 0.3	1.5 ± 0.2	0.040 ± 0.002	3.3 ± 0.3	0.24 ± 0.01	115 ± 5	–20.0 ± 0.8
Gd@C_82_ DMF extract	DM-Probe	0.5 h 10 times	18 ± 2	5.40 ± 0.4	<0.3	2.3 ± 0.3	0.8 ± 0.2	<0.002	<0.001	<0.005	170 ± 5	–23.5 ± 0.9
C_60_Cl_6_	DM-Probe	0.5 h 6 times	16 ± 2^a^	5.80 ± 0.5	<0.3	5.4 ± 0.4	1.5 ± 0.2	<0.002	<0.001	<0.005	180 ± 5	–32.3 ± 0.8
C_70_Cl_10_	DM-Probe	0.5 h 4 times	14 ± 2^a^	3.20 ± 0.3	<0.3	6.8 ± 0.7	1.4 ± 0.2	<0.002	<0.001	<0.005	170 ± 5	–30.3 ± 0.5
C_60_–PCBM	DM-Probe	0.5 h 4 times	20 ± 2^a^	0.7 ± 0.1	<0.3	<0.5	<0.5	<0.002	<0.001	<0.005	80 ± 5	–15.2 ± 0.3
C_60_-Pyrollidin-BHT	DM-Probe	0.5 h 4 times	15 ± 2^a^	0.6 ± 0.1	<0.3	<0.5	<0.5	<0.002	<0.001	<0.005	120 ± 10	–31.3 ± 0.5
Py-C_60_	DM-Probe	0.5 h 4 times	12 ± 2^a^	1.7 ± 0.2	<0.3	<0.5	<0.5	<0.002	<0.001	<0.005	130 ± 15	–30.0 ± 0.8
Water after sonication^b^	DM-Probe	0.5 h 3 times	<0.01^c^	0.4 ± 0.1	<0.3	<0.5	<0.5	<0.002	<0.001	<0.005	n/m	n/m
Water to toluene ratio (1:5 by vol.)^b^	DM-Probe	0.5 h 3 times	0.35 ± 0.09^c^	0.6 ± 0.1	<0.3	3.2 ± 0.3	4.4 ± 0.2	0.010 ± 0.002	0.3 ± 0.1	0.05 ± 0.01	n/m	n/m

n/m, not measured.

a measured by only TOC-analyzer.

b blank (control) experiment.

c estimation of total orgaic carbon in blank (control) experiment without purification by solid-phase extraction of non-polar components.

To estimate the accuracy of analysis (ISO 5725–1:1994), the total organic carbon (TOC) analysis was perfomed. For unmodified products of Gd@C_82_ produced by the sonication technique, the ICP-OES determination was first applied for estimating the concentration through the *endo*-atom. For all the fullerenes used in this study (Table 1), we observed insignificantly different concentrations for samples prepared by solvent exchange (procedure 1) and direct sonication (procedure 2). However, the proposed immersed-probe sonication without organic solvents for AFDs preparation is cleaner, greener, and environmentally friendly.

### 3.3 Impurities

All average concentrations and the total content of impurities in produced AFDs are presented in Table 1, and total metal impurities content are given in Table 2. The presence of these elements as a whole was expected because they are impurity components of the alloy of the ultrasonic probe (see Section 3.3 below).

**Table 2:** Inorganic compositions of aqueous fullerene dispersions samples (*n* = 3, *P* = 0.95) produced by direct sonication.

**Aqueous fullerene dispersion (AFD)**	**Impurities, ppm**						
	**Al**	**B**	**Cr**	**Fe**	**Mo**	**Si**	**Ti**
C_60_	0.10 ± 0.01	0.21 ± 0.03	<0.01	0.15 ± 0.02	<0.01	0.6 ± 0.1	0.6 ± 0.1
C_70_	0.08 ± 0.01	0.34 ± 0.05	<0.01	0.02 ± 0.01	<0.01	0.4 ± 0.1	1.0 ± 0.1
C_60_	0.05 ± 0.01	0.18 ± 0.03	<0.01	0.05 ± 0.01	<0.01	0.6 ± 0.1	0.60 ± 0.1
C_70_	0.10 ± 0.02	0.21 ± 0.02	<0.01	0.06 ± 0.02	<0.01	0.6 ± 0.1	0.5 ± 0.1
Gd@C_82_ DMF extract	0.8 ± 0.1	1.6 ± 0.2	0.05 ± 0.01	0.10 ± 0.02	0.30 ± 0.04	17 ± 1	5.40 ± 0.4
C_60_Cl_6_	0.7 ± 0.1	0.9 ± 0.1	<0.01	0.10 ± 0.02	341	16 ± 1	5.80 ± 0.5
C_70_Cl_10_	0.5 ± 0.1	0.9 ± 0.1	<0.01	0.07 ± 0.02	195	4 ± 1	3.20 ± 0.3
C_60_–PCBM	0.05 ± 0.01	0.6 ± 0.1	<0.01	<0.01	<0.01	1.0 ± 0.2	0.7 ± 0.1
C_60_-Pyrollidin-BHT	0.09 ± 0.02	0.04 ± 0.01	<0.01	<0.01	<0.01	0.10 ± 0.02	0.6 ± 0.1
Py-C_60_	0.13 ± 0.02	0.17 ± 0.02	<0.01	<0.01	0.11 ± 0.01	2.5 ± 0.2	1.7 ± 0.2

In all cases concentrations of elements were not more than; 1 ppb for Ba, Cd, Li, Be; 2 ppb V, Mn, Ni, Cu; 5 ppb As, Co; 10 ppb Ag, Pb, Se, W, Zn; 20 ppb Sb, Sr, Sn; 500 ppb Na, K, Ca, Mg.

Table 1 shows significant differences in the impurity composition of the dispersions depending on the type of technique used but no differences depending on fullerene species. Thus, a solvent-exchange technique results in more impure dispersions with derivatives from organic solvents. As for metal impurities (Table 2), the same composition was found regardless of both the type of technique and fullerene. Thus, as a whole, for all ten fullerenes selected, direct sonication led to comparable results in terms of concentrations of both the main component and impurities. The problems with impurity compositions are described below in sections 3.6 and 3.7.

### 3.4 Fullerene derivatization during the preparation of aqueous dispersions

**Fig. 1:**
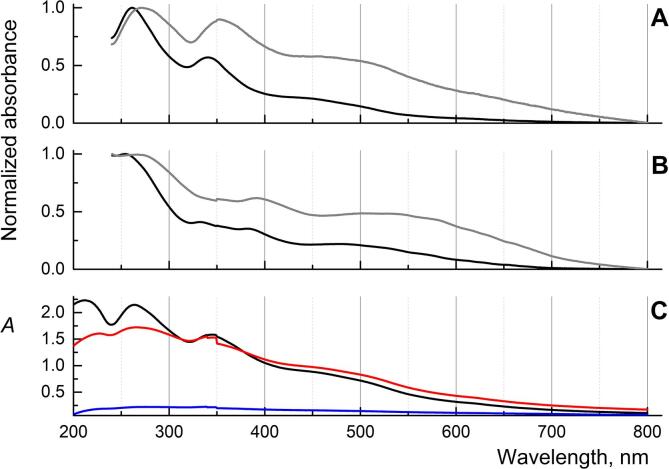
Normalized absorbance spectra of aqueous C_60_ (A) and C_70_ (B) dispersions in the range of 240–800 nm. The concentrations are *c*(C_60_ solvent-exchange technique) = 6.2 ± 0.4 ppm; *c*(C_60_ direct sonication technique) = 4.4 ± 0.5 ppm; *c*(C_70_ solvent-exchange technique) = 5.7 ± 0.7 ppm; *c*(C_60_ direct sonication technique) = 7.4 ± 0.8 ppm. Before spectra registration, samples (A) and (B) were filtered (0.45 μm), the black line for dispersions prepared by the solvent-exchange technique, and the gray line, for direct desperation. Absorbance spectra in the range of 200–800 nm of aqueous fullerene C_60_ pristine (after 15 month of preparation) are *c*(C_60_ direct sonication technique) = 25.0 ± 3.4 ppm (black line), spectra after after 30-min boiling (red line), freezing at –20℃ for 48 h. Ordinate values for: (i) parts A and B are normalized absorbance units from 0 to 1 and (ii) part C are absorbance units. In all cases, the pathlength was 10 mm. Baseline correction made prior the analysis using ultrapure water. (For interpretation of the references to color in this figure legend, the reader is referred to the web version of this article.)

In the case of fullerene derivatives and endohedral fullerenes in aqueous media, there are no significant bands in UV/vis spectra in the range from 200 to 800 nm. Their spectra coincide with the scattering spectrum, as described previously for Y@C_82_ [45]. In the case of C_60_ and C_70_, a comparison of two techniques for AFD preparation shows some differences in the absorption spectra. Red shifts for both AFDs C_60_ and C_70_ have been observed in normalized absorbtion spectra (see. Fig. 1, parts A and B) in the case of direct sonication without toluene. For AFDs absorption maxima shifts were: (1) C_60_ 264 → 270 (6 nm), which may correspond to the residual toluene content interacting with fullerenes; 343 → 355 (12 nm); (2) C_70_ 254 → 273 (19 nm); and 384 → 393 (9 nm). This behavior indicates different intermolecular interactions by close π–π stacking interactions in fullerene clusters and varying H-bond network formation and also, different electrostatic and polarization interactions [54]. The red shift in fullerene absorption spectra is still under discussion and requires a separate study. Still, overall shapes of absorption spectra for C_60_ (direct and solvent-exchange sonication procedures) coincide with the previous studies [12], [13], [43], [44]. In the case of a solvent-exchange procedure for C_70_, the spectrum is also very close to the previous data [43]. For the direct dispersion technique with a probe, a completely different absorption behavior is established. There is a broadening of the spectral lines and a red shift. Also, we observed significant changes in the UV range from 200 to 300 nm for C_70_ (see Fig. 1, part B). This behavior could be associated with the formation of various cluster structures in AFDs, which previously discussed in [12], [22], [54], [55], [56].

MALDI MS techniques allow for the rapid identification state of fullerene in aqueous media, while these colloidal systems' stability question remains an open discussion [43], [45], [56]. The hypothesis of the stabilization of dispersions due to the formation of a network of hydrogen bonds between solvent molecules and fullerene nanoclusters [57] seems likely. Presumably, fullerenes in aqueous media are formed the peculiar nanoclusters.

(i) hydrophobic fullerenes form the core of the cluster, while
(ii) the first-surface layer consists of fullerene oxides, which ensures the stability of the entire dispersion.

**Fig. 2:**
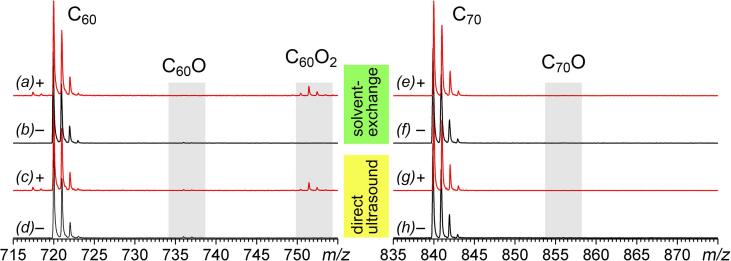
MALDI-TOF-MS spectra of aqueous fullerene dispersions. Spectra (a, c, e, and g) registered in a positive ions mode and (b, d, f, and h) registered in a negative ions mode. Spectra of C_60_ AFD prepared by: (a and b) a solvent-exchange technique; (c and d) direct ultrasound dispergation. Spectra of C_70_ AFD prepared by: (e and f) a solvent-exchange technique; (g and h) direct ultrasound dispergation. The signal of slight modification products is highlighted.

A slight chemical modification from MALDI-MS spectra for AFDs was observed (Fig. 2) for all the studied fullerenes. By relative signal intensity, we estimated the total quantity of C_60_O*_n_* as less *ca.* 1.5 mass.% and C_70_O*_n_*, less *ca.* 0.4 mass.%. Regardless of the dispersion preparation technique, we observed a case-by-case modification. We found the presence of C_60_O and C_60_O_2_ for AFD of C_60_, whereas AFD of C_70_ demonstrates trace amounts of C_70_O; in all other casess, we did not observe a modification, only signals of molecular fullerene ions. MALDI mass spectra of AFDs in the scaled-up regions of 730–780 Da are presented (see Supplementary Information, section S.7). For fullerene derivatives, we observed only signals of molecular ions and did not detect significant signals from hydroxylated fullerene derivatives for C_60_Cl_6_, C_70_Cl_10_, Gd@C_82_, PCBM, Py-C_60_, and C_60_-Pyrollidin-BHT. As well, we demonstrated the form of existence of endofullerenes (the Supplementary Information section S.8).

### 3.5 Stability of aqueous dispersions and their particle-size distribution

For all dispersions, the ionic strength was low, 0.005 M. Zeta-potential values (Table 1) characterize produced AFDs for all test fullerenes as moderately stable [58] and are in good accordance with the data for previously obtained unmodified fullerene nanoparticles [43], [44], [45], [59]. For some samples of AFD C_60_, the stability was also checked for ionic strengths of up to 0.7 M, and the high stability of these suspensions was shown for strengths of <0.05 M [55]. The particle-size distribution is between 80 and 180 nm for aqueous dispersions of both fullerenes and endohedral fullerenes (Table 1). We found no correlations between the nanocluster size and zeta-potential. Unfortunately, the used ultrasonic technique still does not make it possible to regulate particle size in prepared dispersions for a more targeted application. The ultrasound-assisted solvent-exchange technique does not fragment and split nanoclusters to a desired and controllable size. As well, the optimum nanoparticle size depends on the precise site and type of targeted tissues. However, dispersions contaminated with organic surfactants or acquired moieties are of little use for biomedical applications.

In addition, fullerene in AFDs prepared by sonication probe showed long-term storage stability. All produced AFDs have been stable from October 2019 until now (early March 2021). Due to aggregation, the product concentration loss did not exceed 5% for all cases. For example, for C_60_, we have shown stability upon boiling, which is essential for sterilizing solutions. However, with prolonged freezing at –20℃ for 48 h, up to 80% of fullerenes aggregate and precipitate. The corresponding absorption spectra are shown in Fig. 1 (part C). Absorption spectra of AFDs C_60_ underwent boiling displayed an increase in light scattering due to particle aggregation. The particle size distribution (by DLS) has changed from unimodal (no more than 180 nm, Table 1) to bimodal after boiling treatment. The fraction of particles in the bimodal distribution with a size of about 130 nm remains, but a new fraction appears (no more than 10% of the total amount) with a size over 500 nm, since the content of fullerenes in AFDs sharply decreased after defrosting the solution. About 90%, according to the absorption spectra, precipitated and did not undergo further dispersion. It is recommended not to freeze samples during long-term storage and transportation.

### 3.6 Degradation and formation of organic byproducts and metal impurities

**Fig. 3:**
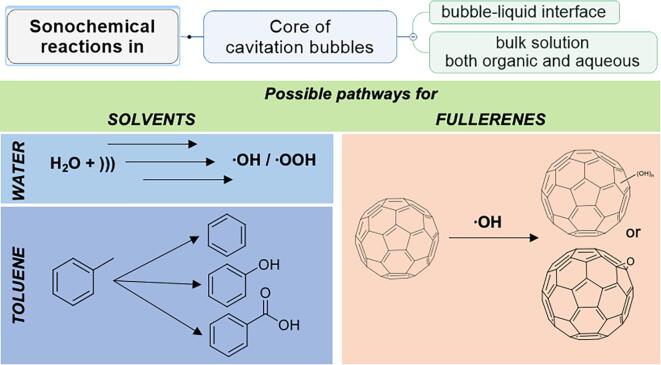
Schematic pathway of possible sonochemical reactions occurring during the preparation of an aquous fullerene dispersion using different tecniques.

A severe drawback of this kind of aqueous dispersions is particles in the raster from the radiolysis of water. Reaction pathways in these systems can be uncontrollable, and some radical formation is non-equilibrium (Fig. 3). The way of sonochemical transformations inside liquid media is the formation to a large extent of hydroxyl radicals. However, the amplitude of ultrasonic waves, external static pressure, temperature, and viscosity of the liquid during sonication influence on the reproducibility [60]. When the acoustic cavitation is on, it produces bubbles with local heating up to 6000 K [61]. The main sonochemical products are H_2_, H_2_O_2_, ${NO}_{3}^{-}$, ${NO}_{2}^{-}$, and NH_3_ [62] but for a low frequency of 20 kHz used in this work, ammonia cannot be formed [63]. In the reaction medium, we have no organic-nitrogen precursor that can lead to NH_3_ and ammonia ion as well.

For the solvent-exchange procedure, sufficiently large amounts of organic solvents are dispersed, and many new compounds are solubilized [64]. Rapid overheating was observed for the toluene–water system, and the solvent (toluene) began to evaporate much faster than in pure water, which may often lead to low yields of AFDs. Therefore, this approach must be abandoned in favor to a direct-mode sonication. Possible pathways of sonochemical reactions for toluene and fullerenes are given in Fig. 3. Shortly, toluene may be transformed into benzene, phenol, and benzoic acid by ultrasound irradiation (Table 1), and fullerenes can slightly obtain epoxy- or hydroxy- moieties. The use of organic solvents entails the further use of sorption purification by solid-phase extraction, which makes approaches more expensive and makes it less “green”. We found the transformation pathway of toluene to benzene, phenol, and benzoic acid, (Fig. 3) by the quantitative analysis of the samples for ultrasound-assisted solvent exchange procedure (Table 1). A similar behavior, the transformation of a neat solvent into organic products for the solvent-exchange procedure with benzene (procedure 4), was not detected.

We purified AFD C_60_ and C_70_ prepared by a solvent-exchange technique. Before SPE, the total content of toluene was *ca.* a few units of ppm after sorption the entire content was less 10 ppb, loss of fullerenes amounted to 10% (estimated by absorbance). Thus, the efficiency of the purification procedure, which can be used in conjunction with the methodology for preparation dispersions, is shown.

### 3.7 Probe-decomposition contamination

Sample homogenization with an immersed ultrasound probe was more efficient than with an ultrasound bath due to the large amplitude of the ultrasonic waves. Due to high-energy acoustic cavitation, the material of the probe is decomposed [61]. The first drawback is that the treated solutions are enriched by titanium liberated from the eroded probe (Table 2). It is almost an intractable drawback of this technique as filtration does not complete remove titanium from solution [65]. Previously, the total content level of these elements in dispersions is not critical for all the fullerenes studied. We assume that the form of existence of these elements in dispersion is either hydrated metal complexes or colloidal nanoparticles. However, the study of metal species was beyond the scope of this work. Such a metal content confines the possible use of dispersions *in vitro* or *in vivo*; however, it is necessary to purify the aqueous dispersions before, e.g., a clinical trial.

**Fig. 4:**
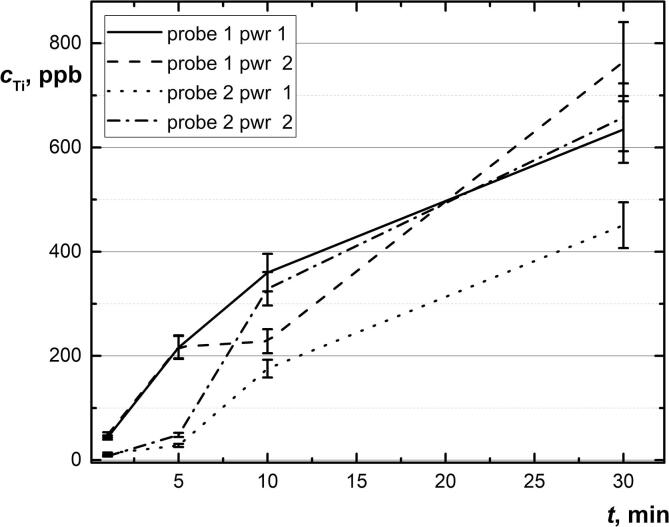
Average values of total titanium measured by ICP-OES during immersed-probe sonication of ultrapure water at different expose times. Probe 1 (with a larger surface area); Probe 2 (with less surface area) at different electrical power 0.3 and 0.6 kW. (*n* = 3, *P* = 0.95).

As well, the kinetic data of probe decomposition in two electrical power modes and different types of the probe were obtained. The total titanium content increases with solution exposure. After 5 min of treatment for an ultrasonic probe with a working surface area of 6.6 cm^2^, and 10 min for a probe with a smaller surface area of 0.6 cm^2^, the maximum permissible concentration of titanium, 100 ppb [66] is exceeded (Fig. 4). More complete data on metal content are given in Supplementary data (section S.1, Table S2).

Moreover, as a general rule, the time needed to perform an analytical application (e.g., solid–liquid extraction) with an ultrasound probe is 1 to 3 min [19] then the sonication device (probe) is degraded. In the case of fullerene dispersions, this time is too low. Thus, we recommend using a sonication device with low amplitudes (practically, a smaller probe working area) and a low electrical power to avoid rapid and uncontrollable worsening of ultrasonic transducers.

### 3.8 Concentrations of pristine fullerenes C_60_ and C_70_ in aqueous dispersions

Table 3 compares best reported concentrations of C_60_ and C_70_ in AFDs. These concentrations were obtained using direct (DM) or solvent-exchanged ultrasound-assisted (SE-US or SE-probe) procedures. It is noteworthy that DM- and SE-probe produce almost equal concentrations for C_60_ and C_70_. An explanation of this is that a high-power probe treatment rapidly evapores of toluene. In this case, SE-probe is reduced to DM-probe procedure for both fullerenes. As it is seen from the Table 3, SE-US give a certain increase of concentration of C_60_ compared to direct procedures with both probe, and conventional mild US technique. The quasi-equilibrium model [43] for SE-US predicts even higher concentrations of C_60_ and C_70_, up to 500 ppm. Thus, to achieve the maximum amount of fullerenes in an aqueous dispersion, SE-US should be preferred to a direct procedure. Table 3, however, shows that experimental concentrations of the fullerenes differed only slightly, and using of a probe technique does not lead to a visible loss of fullerene content.

**Table 3:** Concentrations of C_60_ and C_70_ in aqueous dispersions. Concentration measured by total organic carbon analysis (*n* = 3, *P* = 0.95); average yield in all conducted experimenrts.

**Fullerene**	**Type of AFD**	**Type of sonicator**	**The average concentration of fullerenes, ppm (*n* = 3, *P* = 0.95)**	**Average yield, % (*n* = 5, *P* = 0.95)**	**Reference**
C_60_	DM-probe	probe	68 ± 2	66 ± 6	This study
C_60_	SE-probe		72 ± 4	65 ± 7	
C_70_	DM-probe		62 ± 3	55 ± 8	
C_70_	SE-probe		68 ± 3	52 ± 7	
C_60_	SE-US	bath	129.6	54	[43]
C_70_	SE-US		58	45	[43]
C_60_	D-US		11.6	n/m^a^	[75]

a , not mentioned.

**Table 4:** Ultrasound bath or probe to fullerene dispersions preparation: pros and cons.

**Technique**	**Ultrasound source**	**Loading capacity, sonication efficiency**	**Reproducibility**	**Yield**	**Time-saving**	**Money-saving**	**Eco-friendly**	**Rate of fullerene surface modification**	**Need for**	
									**purification**	**filtration**
**Solvent-exchange (toluene)**	Bath	+	++	++	–	–	– –	++	slightly	is required
**Direct sonication**		+	+	–	– –	+	+	+++	slightly	
**Solvent-exchange (toluene)**	Probe	+++	+++	+++	++	+	– –	+	total	
**Direct sonication**		+++	+++	+++	++	–	++	+	slightly	
**Advantages of a sonication probe and some peculiarities for fullerenes**		Due to higher supplied acoustic energy and variation of probe types (length, shapes, working area)	In the probe mode, a reproducible sonicator immersion occurs	Due to higher system temperature during the preparation and higher total supply acoustic energy [76]	The same as for the «Yield» [77]	Disadvantages due to further specific purification steps	Due to using a neat organic solvent, such techniques are less preferred [78]	Due to a lower exposure time, ultrasonic probes produce fewer modifications (akin epoxides etc.)	To reduce organic and inorganic species	Always needs to reduce oversized agglomerates (over ~ 450 nm and higher) by syringe membrane filtering.

**+++, ++,** efficiently, use justified

**+,** effective if we neglect the purity of the prepared dispersions, not suitable for medical purposes

**–,** alternative solutions are required; if necessary, you can use

**– –,** it is reasonable to refuse to use

### 3.9 Advantages of using sonication probe in fullerene dispersion preparation

The benefits of using an ultrasonic probe over an ultrasonic bath, (particularly a cleaning bath), have been described previously [18], [19]. However, these statements are formulated in generic terms of reproducibility and precision and require additional clarification for the synthesis of carbon nanomaterials. As a result of this study, we can formulate recommendations for the use of aqueous fullerene dispersions in the synthesis Table 4. Ultrasonic probes have the principal advantages of delivering energy more precisely. However, we need to bear in mind to check the purity of the obtained products for further applications. In general, ultrasonic probes show a great performance in loading capacity in synthesis, total yield, acceptable final product concentration timesaving, and lower filtration steps. In our opinion, ultrasound probes help synthesizing AFDs by (1) high-power ultrasound frequency (22 kHz; default, not changeable values for applied equipment); (2) a high amplitude of ultrasonic waves; (3) a wide intensity range (up to 250 W/cm^2^, which regulated by replaceable tips with different working areas). As a whole, it decreases the exposure time and reduces the number of contaminant components, while the product yield has not changed.

## 4 Conclusions

Thus, we have proposed a green-chemistry approach for preparing aqueous dispersions of fullerenes C_60_, C_70_, their derivatives phenyl-C_61_-butyric acid methyl ester, C_60_Cl_6_, C_70_Cl_10_, pyrollidinofullerene bearing two pyridyl groups, C_60_-Pyrollidin-BHT, and Gd@C_82_ by direct ultrasonication with an immersed ultrasound probe, which is waste-free and does not involve organic solvents, thus, is cost-effective and sustainable. We have achieved concentrated, pure enough, and more than 18-month stable aqueous fullerene dispersions of ten different fullerenes and made their elemental, inorganic, and organic characterization. In general, AFDs prepared using ultrasonic baths remain the purest and contain only the components of the process-container glass flask [44]. However, in the case of an immersed probe, we have:

(i) only probe impurities like Ti and other elements (Tables 1 and 2)
(ii) only a slight transformation of fullerenes to C_60_O, C_60_O_2_, etc.
(iii) no organic byproducts. The use of a high-powered ultrasonic device contributes to an increase in the yield of fullerene dispersions; moreover, we believe that such an approach gets to work with aqueous solutions of other carbon materials.

Further development of this approach may lead to a larger (industrial) scale of AFD production. Ultrasound-assisted synthesis of dispersions for industrial production on a large scale has already been demonstrated [26]. However, in the case of AFDs, probe contamination should be removed entirely. Preparation of AFDs on a large scale may decrease total impurities of the dispersions, reduce production costs, and improve the batch-to-batch purity. The following approaches to purification can be considered for AFDs:

(i) dialysis or electrodialysis methods [67]
(ii) electrolyte adding for coagulation [68]
(iii) adding an anticoagulant medication (like enoxaparin sodium [69], [70]) which is used to avoid deep vein thrombosis and pulmonary embolism [71]. Unfortunately, the best approach is not clear yet.

From the viewpoint of the underlying mechanism, phenomenological thermodynamic models of (quasi-) equilibria in aqueous dispersion synthesis should be developed to understand the stability of AFDs. First approaches and concepts have been provided to study ultrasound-assisted extraction, which correlate with the AFD production process [72]. Also, mechanisms of fullerene stability in aqueous media should be elucidated; the first steps for organic solutions have already been taken [73] claiming the formation of fullerene anion-radicals and/or electron transfer from the solvent. Further potential applications for AFD synthesized by the proposed procedure may deal with targeted, sustained antibacterial therapies [74]. The design of experimental conditions using an ultrasound probe can help achieving quantitative yields in preparing dispersions, which is crucial for very expensive endofullerenes. Large-scale synthesis can be successfully conducted. Also, approaches to complete purification of such aqueous dispersions should be improved or proposed.

## CRediT authorship contribution statement

**Ivan V. Mikheev:** Funding acquisition, Investigation, Methodology, Writing - original draft. **Mariya O. Pirogova:** Investigation. **Liliia O. Usoltseva:** Investigation. **Anna S. Uzhel:** Investigation. **Timofey A. Bolotnik:** Investigation. **Ivan E. Kareev:** Investigation, Recourses. **Viacheslav P. Bubnov:** Investigation, Recourses. **Natalia S. Lukonina:** Investigation, Recourses. **Dmitry S. Volkov:** Conceptualization. **Alexey A. Goryunkov:** Writing - review & editing. **Mikhail V. Korobov:** Writing - review & editing. **Mikhail A. Proskurnin:** Writing - review & editing, Conceptualization, Supervision.

## Declaration of Competing Interest

The authors declare that they have no known competing financial interests or personal relationships that could have appeared to influence the work reported in this paper.
