# Supplementary material for: Green and rapid preparation of long-term stable aqueous dispersions of fullerenes and endohedral fullerenes: The pros and cons of an ultrasonic probe
Source: Ultrason Sonochem. 2021 Mar 19;73:105533. doi: 10.1016/j.ultsonch.2021.105533 (PMC8044700; doi:10.1016/j.ultsonch.2021.105533)
Supplement: Supplementary data 1 [file mmc1.docx]

Supporting Information

**Green and rapid preparation of long-term stable aqueous dispersions of fullerenes and endohedral fullerenes: The pros and cons of an ultrasonic probe**

by

I.V. Mikheev^a,*^, M.O. Pirogova^a^, L.O. Usoltseva^b^, A.S. Uzhel^a^, T.A. Bolotnik^a^, I.E. Kareev^c^, V.P. Bubnov^c^_,_ N.S. Lukonina^b^, D.S. Volkov^a^, A.A. Goryunkov^b^, M.V. Korobov^b^, and M.A. Proskurnin^a^

^a^ *Chemistry Department Analytical Chemistry Division of Lomonosov Moscow State University 119991 Moscow, Russia*

^b^ *Chemistry Department Physical Chemistry Division of Lomonosov Moscow State University 119991 Moscow, Russia*

^c^ *Institute of Problems of Chemical Physics of the Russian Academy of Sciences 142432 Chernogolovka, Moscow Region, Russia*

^*^Corresponding author. E-mail address: mikheev.ivan@gmail.com, tel: +74959391568

Table of Content

[S.1 Conditions of cations content measurements by ICP-OES (Inductively coupled plasma atomic emission spectroscopy) 3](#_Toc65596576)

[S.2 Conditions of anions content measurements by IC (Ion chromatography) 5](#_Toc65596577)

[S.3 Conditions of volatile and non-volatile organic content measurements by Headspace GC/MS and HPLC-FLD and HPLC-UV (Headspace Gas Chromatography/Mass Spectrometry and High-performance liquid chromatography with fluorescence and UV detection) 8](#_Toc65596578)

[S.4 Characteristics of the ultrasonic probe device and operating modes 16](#_Toc65596579)

[S.5 Photos of the ultrasonic probe (tips) with the different operating areas 17](#_Toc65596580)

[S.6 Fullerene derivatives description 18](#_Toc65596581)

[S.7 MALDI MS spectra 20](#_Toc65596582)

[S.8 Forms of existence of endohedral fullerenes in aqueous dispersions 21](#_Toc65596583)

1. Conditions of cations content measurements by ICP-OES (Inductively coupled plasma atomic emission spectroscopy)

All measurements were performed by ASTM D5673–16.

| Table S. 1. ICP-AES operating parameters | |
| --- | --- |
| RF power, kW | 1.3 |
| Nebulizer gas flow rate, L/min | 0.95 |
| Auxiliary gas flow rate, L/min | 1.5 |
| Plasma gas flow rate, L/min | 18 |
| Sample flow rate, rpm | 12 |
| Integration time, s | 25 |
| Replicates | 3 |

| Table S2 Metal content for sonication probe decomposition at varying time and electrical power | | | | | | | | | | | |
| --- | --- | --- | --- | --- | --- | --- | --- | --- | --- | --- | --- |
| Time of sonication probe exposes, min | Sonication horn surface area, cm^2^ | Electrical power, W | Impurities, ppb | | | | | | | | |
|  |  |  | Al | B | Cr | Fe | Mo | Si | Ti | Zn |  |
| 1 | 6.605 | Mode 1 – 300 | <20 | <10 | <10 | <10 | <5 | 23 ± 2 | 43 ± 4 | 14 ± 1 |  |
| 5 |  |  | <20 | <10 | <10 | <10 | 10 ± 1 | <20 | 216 ± 22 | 3 ± 1 |  |
| 10 |  |  | 23 ± 2 | <10 | <10 | <10 | 19 ± 2 | <20 | 360 ± 36 | 2 ± 1 |  |
| 30 |  |  | 42 ± 4 | <10 | 11 ± 1 | <10 | 32 ± 3 | 35 ± 4 | 635 ± 64 | 3 ± 1 |  |
| 60 |  |  | 97 ± 9 | 47 ± 5 | 24 ± 2 | 17 ± 1 | 52 ± 5 | 31 ± 3 | 1508 ± 150 | 3 ± 1 |  |
| 120 |  |  | 91 ± 9 | 37 ± 4 | 19 ± 2 | 26 ± 2 | 41 ± 4 | 33 ± 3 | 1214 ± 120 | 48 ± 5 |  |
| 1 |  | Mode 2 – 600 | <20 | <10 | <10 | <10 | <5 | 27 ± 3 | 48 ± 5 | 3 ± 1 |  |
| 5 |  |  | <20 | <10 | <10 | <10 | 10 ± 1 | 26 ± 3 | 217 ± 22 | 4 ± 1 |  |
| 10 |  |  | <20 | <10 | <10 | <10 | 10 ± 1 | <20 | 228 ± 22 | 9 ± 1 |  |
| 30 |  |  | 52 ± 5 | 44 ± 4 | 13 ± 1 | 32 ± 3 | 29 ± 3 | 39 ± 4 | 765 ± 78 | 2 ± 1 |  |
| 60 |  |  | 24 ± 3 | 40 ± 4 | <10 | 18 ± 2 | 12 ± 1 | 33 ± 3 | 347 ± 35 | 5 ± 1 |  |
| 120 |  |  | <20 | 30 ± 3 | <10 | 19 ± 2 | 10 ± 1 | 45 ± 4 | 245 ± 24 | 11 ± 1 |  |
| 1 | 0.628 | Mode 1 – 300 | <20 | 26 ± 3 | <10 | <10 | <5 | <20 | 12 ± 2 | 2 ± 1 |  |
| 5 |  |  | <20 | 22 ± 2 | <10 | <10 | <5 | <20 | 28 ± 3 | 3 ± 1 |  |
| 10 |  |  | <20 | 20 ± 2 | <10 | <10 | 9 ± 1 | <20 | 176 ± 20 | 2 ± 1 |  |
| 30 |  |  | 39 ± 4 | 19 ± 2 | <10 | 7 ± 1 | 16 ± 2 | 33 ± 2 | 451 ± 45 | 8 ± 1 |  |
| 1 |  | Mode 2 – 600 | <20 | 24 ± 2 | <10 | <10 | <5 | <20 | 8 ± 1 | 2 ± 1 |  |
| 5 |  |  | <20 | 22 ± 2 | <10 | <10 | <5 | <20 | 48 ± 4 | 3 ± 1 |  |
| 10 |  |  | <20 | 19 ± 2 | <10 | <10 | 13 ± 1 | <20 | 329 ± 33 | 3 ± 1 |  |
| 30 |  |  | 43 ± 4 | 18 ± 2 | 11 ± 1 | <10 | 31 ± 3 | <20 | 658 ± 75 | 4 ± 1 |  |
| In all cases concentrations of elements were not more than 1 ppb for Ba, Cd, Li, Be; 2 ppb V, Mn, Ni, Cu, 5 ppb As, Co; 10 ppb Ag, Pb, Se, W; 20 ppb Sb, Sr, Sn; 500 ppb Na, K, Ca, Mg | | | | | | | | | | | |

1. Conditions of anions content measurements by IC (Ion chromatography)

All measurements were performed by EPA Method 300.1, Revision 1.0.

Eluent — 1.4 mM NaHCO_3_/1.5 mM Na_2_CO_3_; Flow rate — 2.0 ml/min. An isocratic elution mode was used. The injected volume of the sample is 25 μl. Standard anion solutions were prepared by diluting mixtures of anion SRM in a 100.0 ml volumetric flask and subsequent dilution. Retention times (min) of anions were 1.02, fluoride; 1.20, chloride; 1.71, nitrate; 3.11, sulfate; 3.67, phosphate.

To prevent uncontrolled precipitation of the sample during analysis and sorption on the column of fullerene nanoparticles, the sample was centrifuged (180 min, 4 krpm) and passed through a microporous filter (0.22 μm) before the analysis.

| 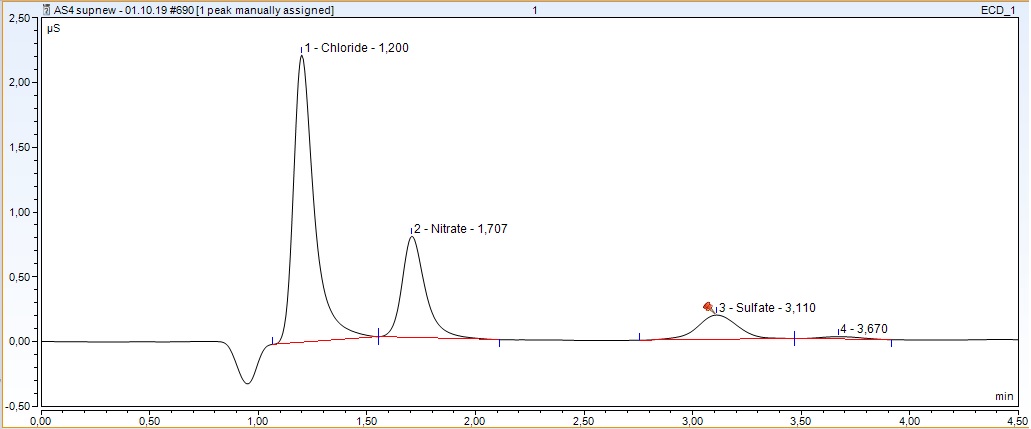 |
| --- |
| Figure S. 1 Chromatogram of aqueous dispersions of С_60_ produced by an ultrasound-assisted solvent exchange procedure. Column: Dionex IonPac AS4 4×250 mm. Precolumn: Dionex IonPac AG4 4×50 mm. Flow rate, 2.0 mL/min. |
| 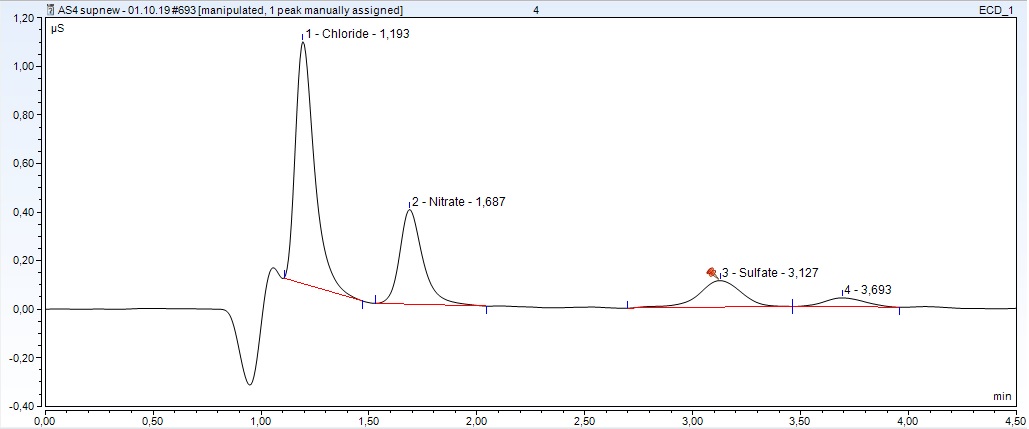 |
| Figure S2 Chromatogram of aqueous dispersions of С_70_ produced by ultrasound-assisted solvent exchange procedure. Column: Dionex IonPac AS4 4×250 mm. Precolumn: Dionex IonPac AG4 4×50 mm. Flow rate, 2.0 mL/min. |
| 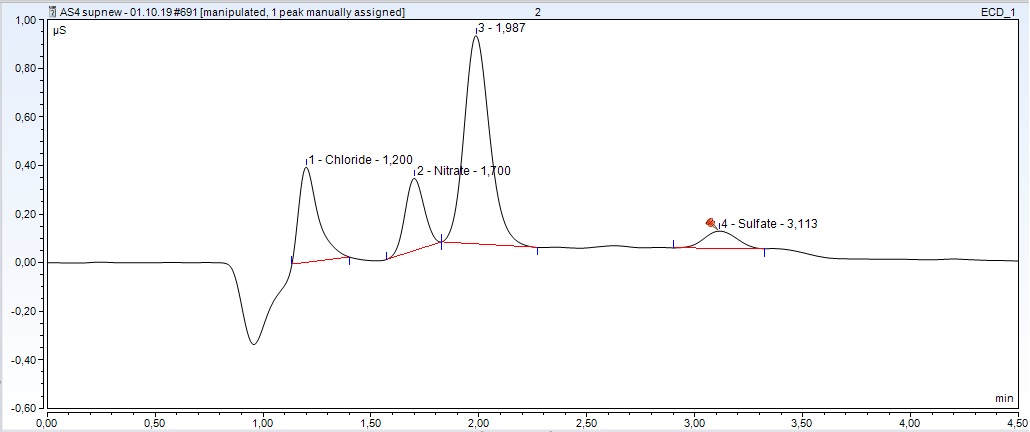 |
| Figure S. 3 Chromatogram of aqueous dispersions of С_60_ produced directly by sonication procedure. Column: Dionex IonPac AS4 4×250 mm. Precolumn: Dionex IonPac AG4 4×50 mm. Flow rate, 2.0 mL/min. |
| 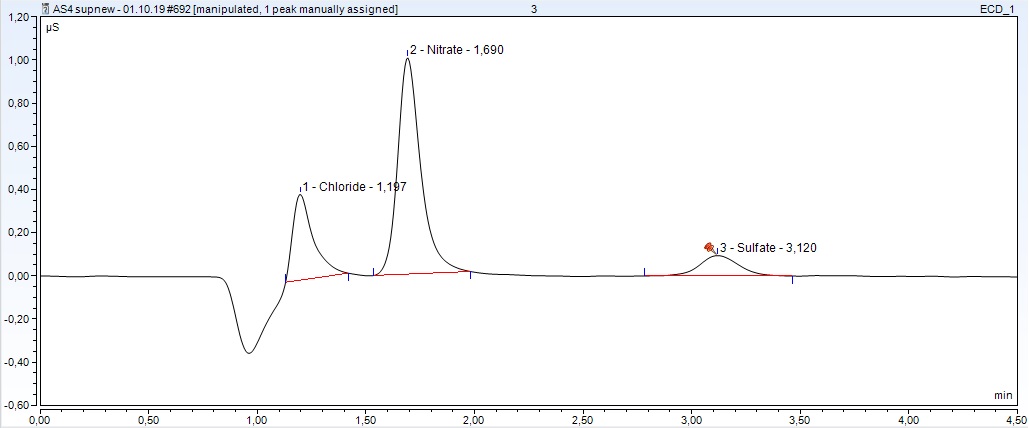 |
| Figure S. 4 Chromatogram of aqueous dispersions of С_70_ produced directly by sonication procedure. Column: Dionex IonPac AS4 4×250 mm. Precolumn: Dionex IonPac AG4 4×50 mm. Flow rate, 2.0 mL/min. |

1. Conditions of volatile and non-volatile organic content measurements by Headspace GC/MS and HPLC-FLD and HPLC-UV (Headspace Gas Chromatography/Mass Spectrometry and High-performance liquid chromatography with fluorescence and UV detection)

All measurements were performed by ASTM D3871-84(2017) for headspace Gas Chromatography/Mass Spectrometry and Internal techniques having used in the laboratory for phenol content by HPLC-FLD.

**Volatile Organic Compounds (VOCs) determination by Headspace Gas Chromatography/Mass Spectrometry**

A 5 ml of sample aliquot was placed in a vial for headspace analysis, hermetically sealed and thermostated for 20 min at 70°C, then 1 ml of the vapor phase was introduced into the chromatograph and analyzed according to the conditions presented in Table S3.

Table S3. Conditions of headspace Gas Chromatography/Mass Spectrometry analysis

| Column | Agilent СP-5 Sil (30 m×0.25 mm×0.4 μm) |
| --- | --- |
| Glass liner | Helium |
| Flow rate | 1.0 ml/min |
| High Pressure Injection | 1:1 |
| Injection Temp. | 250°С |
| Interface Temp. | 200°С |
| Column Oven Temp | 40°C (5min) → 10°C/min → 90°C (5min) |
| Ionization Energy | 70 eV |
| Source temperature | 200°С |
| m/z (selected ion mode) | 78, 91, 104, 106 |

| 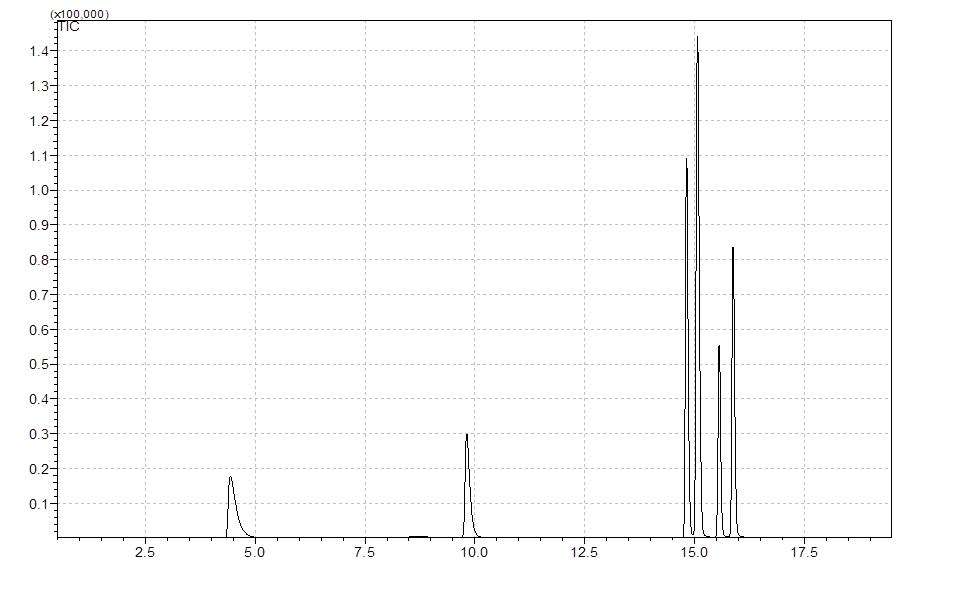 |
| --- |
| Figure S. 5 Chromatogram of a standard reference material of Volatile Organic Compounds 0.12 ppm. (Total Ion Current). The retention time of components increases in a sequence of benzene, toluene, ethylbenzene, and the sum of *m-* and *p-*xylene, styrene, and *o-*xylene. |
| **** |
| Figure S. 6 Chromatogram of aqueous dispersions of С_70_ produced by an ultrasound-assisted solvent-exchange procedure. The peak position is in good accordance with the toluene retention time. *c*_Toluene_=3.3 ppm |

**Phenol content by HPLC-FLD**

The work was carried out according to the method based on the analysis of the sample by HPLC with an FLD (DAD) detector. The concentration of the substance in the sample was calculated using the analysis of standard phenol solutions, taking into account the linear dependence of the chromatographic peak area on the substance contained in the calibration solution, taking into account the weight of the sample and the volume of the sample.

**Sample preparation before analysis**

The sample was filtered through a Whatman® quantitative filter paper (ashless, Grade 589/2 white ribbon) and then through a membrane filter (Cellulose Acetate CA Syringe Filters, 0.45 μm, 25 mm). 1 ml of the prepared sample was transferred to a vial for analysis.

Table S4. Conditions of phenol content analysis by HPLC-FLD

| Column / precolumn | Synergi Hydro-RP (Phenomenex, USA) 250×4.6 mm in size, sorbent particle diameter 4 μm / Security Guard C18 (4×3 mm; Phenomenex, USA); |
| --- | --- |
| Column temperature | 30°С |
| Speed of the mobile phase | 1 ml/min |
| Mobile phase | Eluent A — water (55% vol.) |
|  | Eluent B — acetonitrile (45% vol.) |
| Fluorescent detection conditions | At excitation wavelength — 275 nm and emission wavelength — 315 nm. |
| Retention time (phenol) | 4.9 ± 0.1 min |
| The volume of the injected sample | 100 μL |

The chromatograms of the samples are shown below.

| 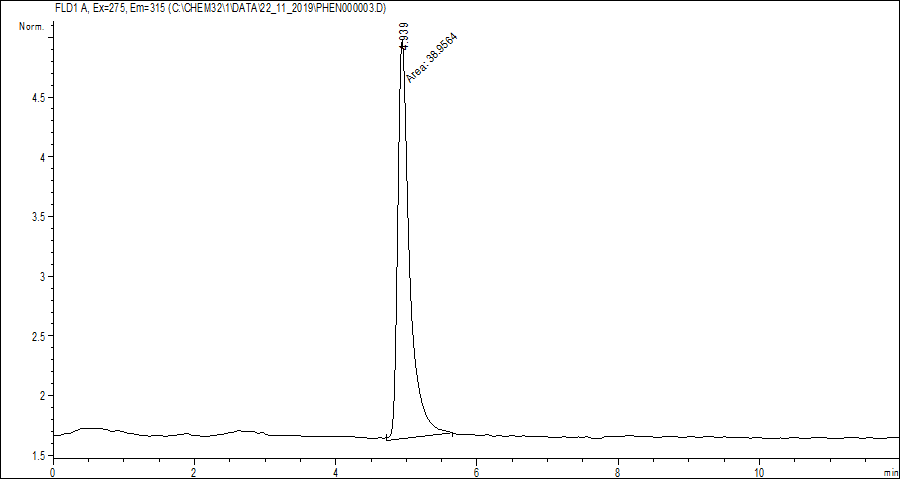 |
| --- |
| Figure S7 Chromatogram of a standard reference material of phenol 0.100 ppm. |
|  |
| Figure S. 8 Chromatogram of a standard reference material of phenol 0.002 ppm. |
|  |
| Figure S. 9 Chromatogram of Chromatogram of aqueous С_70_ dispersions produced by an ultrasound-assisted solvent exchange procedure. The peak position is in good accordance with phenol retention time *c*_Phenol_=0.04 ppm |

**Benzoic acid content by HPLC-UV**

This part of the work was carried out according to the method based on the analysis of the sample by HPLC with a UV detector. The concentration of the substance in the sample was calculated using the analysis of standard solutions of benzoic acid taking into account the linear dependence of the chromatographic peak area on the substance contained in the calibration solution and the weight of the sample and the volume of the sample.

**Sample preparation before analysis**

The sample was filtered through a Whatman® quantitative filter paper (ashless, Grade 589/2 white ribbon) and then through a membrane filter (Cellulose Acetate CA Syringe Filters, 0.45 μm, 25 mm). 1 ml of the prepared sample was transferred to a vial for analysis.

Table S5. Conditions of phenol content analysis by HPLC-UV

| Column / precolumn | Eclipse XDB-C18 (150×4.6 mm, Agilent, USA) / Security Guard C18 (4×3 mm; Phenomenex, USA); |
| --- | --- |
| Column temperature | 30°С |
| Speed of the mobile phase | 1 ml/min |
| Mobile phase | Eluent A — 0.2 mass.% H_3_PO_4_ (83% vol.) |
|  | Eluent B — acetonitrile (17% vol.) |
| Fluorescent detection conditions | At emission wavelength — 235 nm, spectral bandwidth — 4 nm |
| Retention time (phenol) | 11.3 ± 0.1 min |
| The volume of the injected sample | 100 μL |

The chromatograms of the samples are shown below.

|  |
| --- |
| Figure S10 Chromatogram of a standard reference material of benzoic acid 0.017 ppm. |
|  |
| Figure S. 11 Chromatogram of a standard reference material of benzoic acid 0.170 ppm. |
|  |
| Figure S12 Chromatogram of Chromatogram of aqueous С_70_ dispersions produced by an ultrasound-assisted solvent exchange procedure. 2-fold diluted sample. The peak position is in good accordance with benzoic acid retention time *c_Benzoic acid_*=0.24 ppm. |

1. Characteristics of the ultrasonic probe device and operating modes

| **Parameter** | **Value** |
| --- | --- |
| The intensity of ultrasonic exposure, W/cm^2^ | up to 250 |
| Switching power, W | 600 |
| Operating frequency, kHz | 22 |
| Continuous operation, hour | 8 |
| Unit power, V, Hz | 200—230, 50 |

1. Photos of the ultrasonic probe (tips) with the different operating areas

| 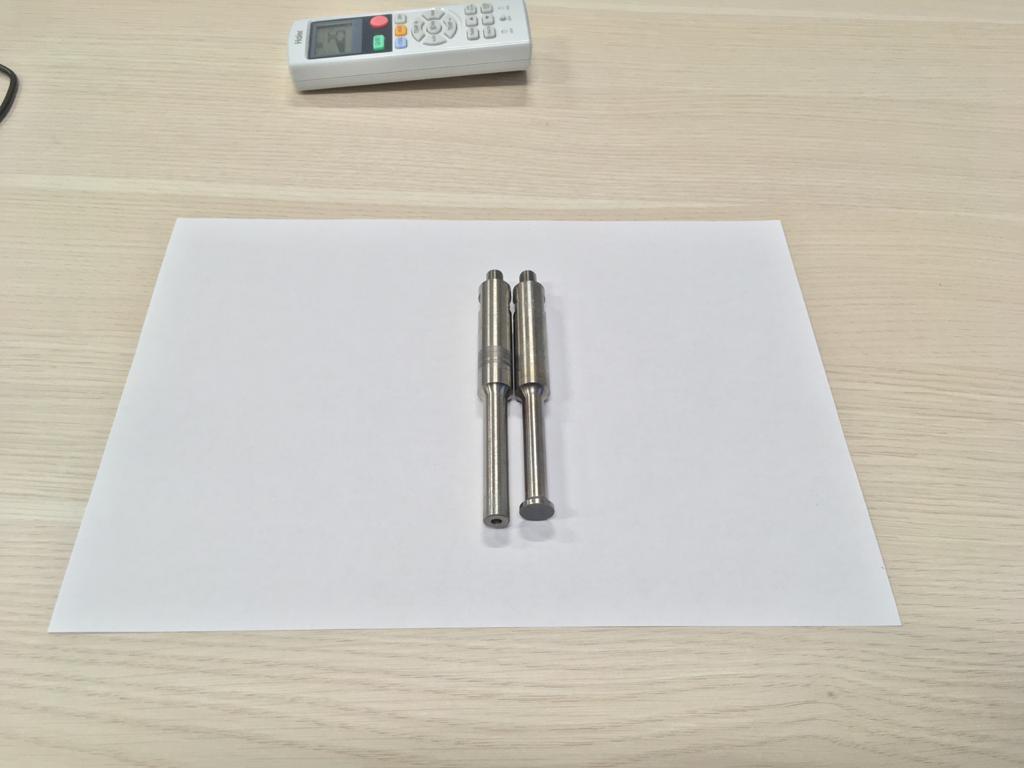 | 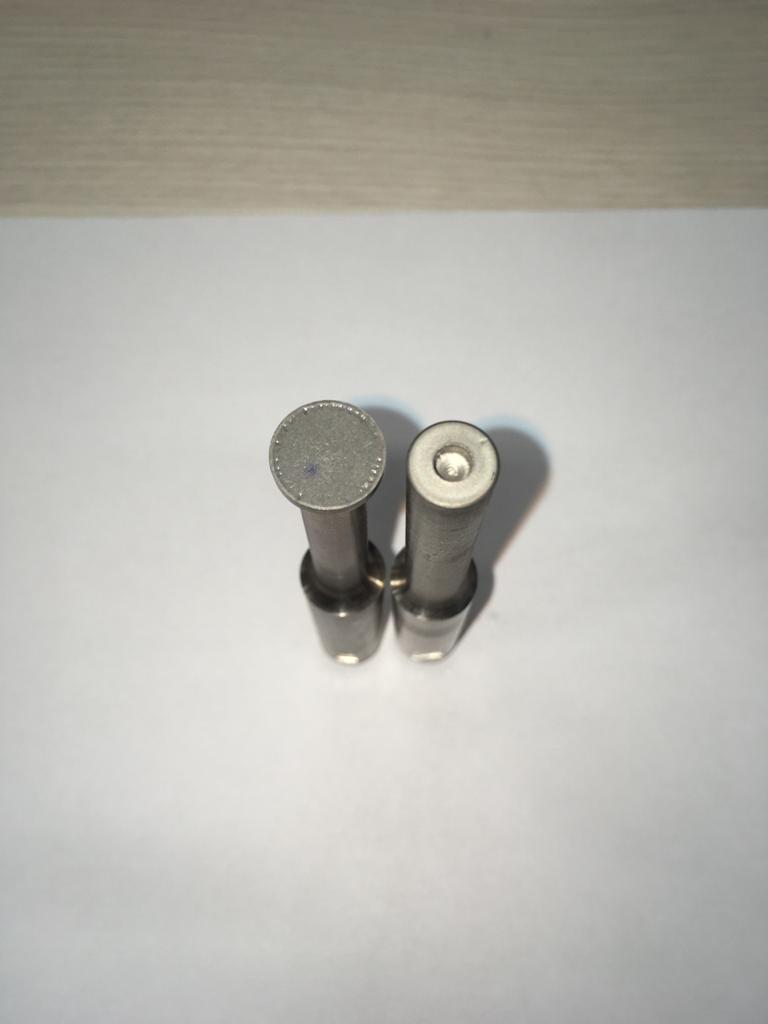 |
| --- | --- |
| **A** | **B** |
| Figure S13 Ultrasound probe tips used for aqueous dispersion preparations. A, side view and B, working area view. The working surface of the probe is marked with a red circle. The sonication efficiency depends on working probe area. | |

1. Fullerene derivatives description
2. The sample of C_60_Cl_6_ was synthesized as described in [1].
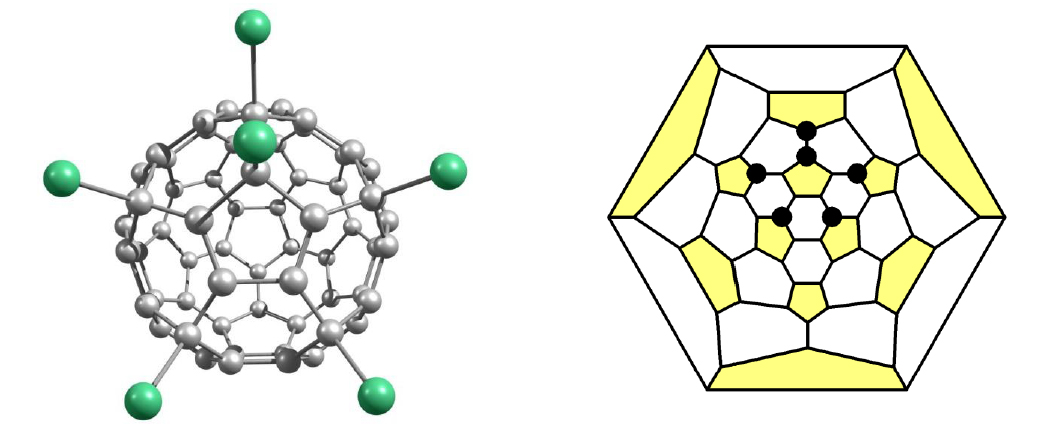


Figure S14 Schlegel diagram and structure of C_60_Cl_6_.

1. The sample of C_70_Cl_10_ was synthesized as described in [2].


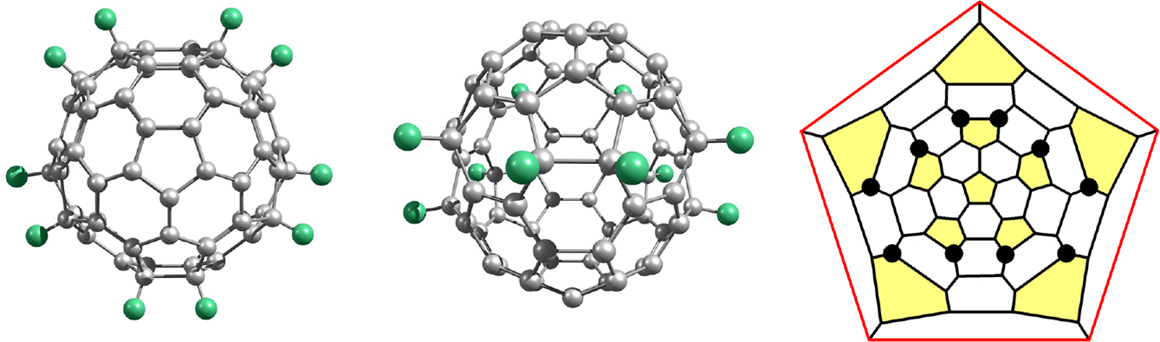


Figure S15 Schlegel diagram and structure of C_70_Cl_10_

| 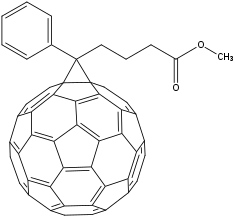  Figure S16 Structure of C_60_–PCBM | 1. C_60_–PCBM. Commercially available PCBM (Phenyl-C_61_-butyric acid methyl ester) powder was used (Nano-C, 99%) |
| --- | --- |
| *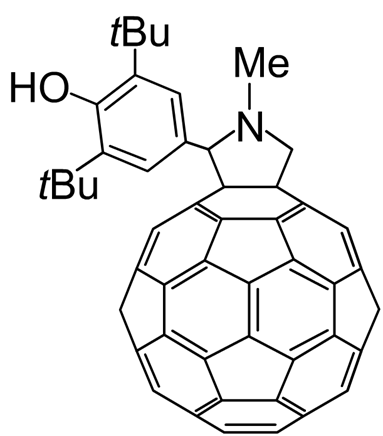*  Figure S17 Structure of C_60_-Pyrollidin-BHT | 1. N-methyl-2-[3,5-di-tert-*butyl*-4-hydroxyphenyl]pyrrolidine fullerene derivatives (C_60_-Pyrollidin-BHT) [3] |
| 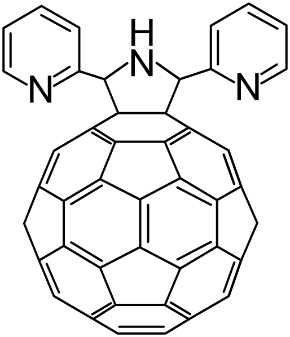  Figure S18 Structure of Py-C_60_ | 1. Pyrollidinofullerene bearing two pyridyl groups (Py-C_60_) derivative was obtained by the Prato reaction [4]. |

1. MALDI MS spectra

Additional information about fullerene state in AFDs presented below.

C_60_O and C_70_O were observed as trace admixtures in MALDI MS spectra of AFD prepared via both techniques. Additional figures of the MALDI MS spectra with enlarged regions of fullerene oxide ions.


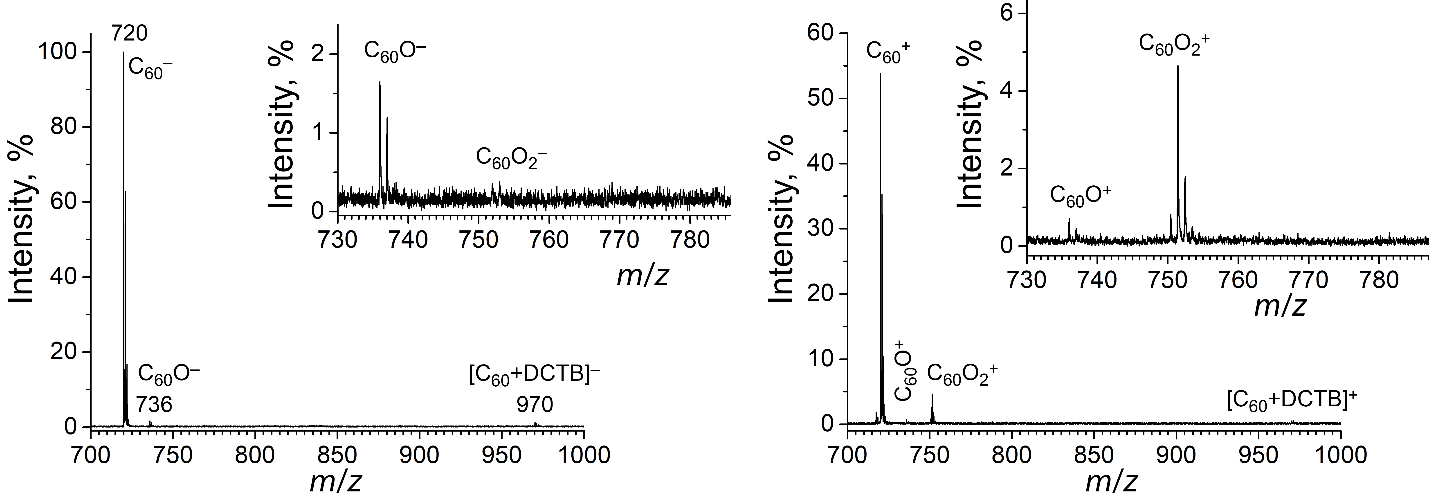


Figure S19. MALDI mass spectra of negative (left) and positive (right) ions of the C_60_ AFD obtained via solvent exchange method. The scaled-up regions of 730–780 Da are presented on the insets.


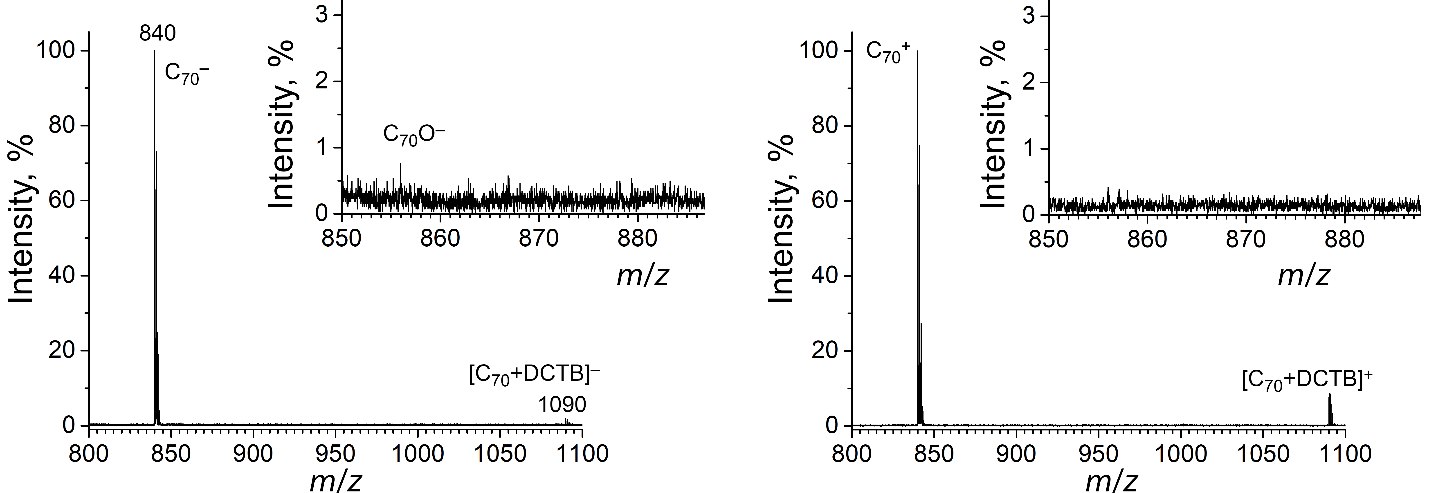


Figure S20. MALDI mass spectra of negative (left) and positive (right) ions of the C_70_ AFD obtained via a solvent exchange method. The scaled-up regions of 850–890 Da are presented on the insets.

1. Forms of existence of endohedral fullerenes in aqueous dispersions

**
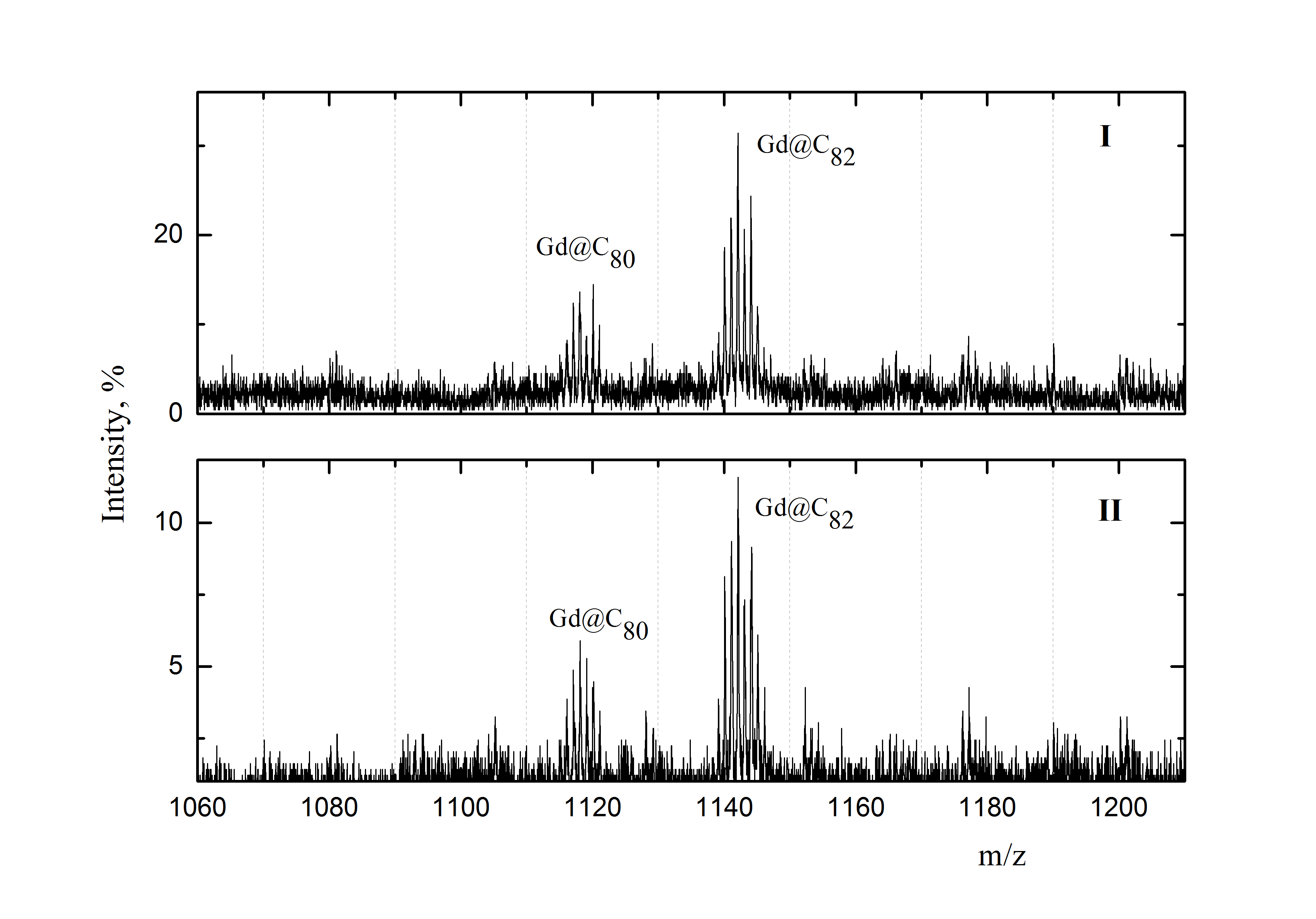
**Figure S21. MALDI mass spectra of (I) suspension of solid enriched extract pristine endofullerene Gd@C_80_/Gd@C_82_, (II) AFDs of endofullerene.

For AFDs Gd@C_80_/Gd@C_82_ in water, the ratio of peak intensities in the mass spectra for the enriched solid extract and AFDs sustains at a level of 1:2, which indicates an equilibrium process of solubilization and the transfer of fullerenes to AFDs with preservation of the ratio of fullerene forms (figure S.21).

After the transfer of fullerenes from metallofullerene-enriched (Gd@C_80_, Gd@C_82_) solid extracts or HPLC-pure samples in toluene (Gd@C_80_, Gd@C_82_), the question arises about the forms of existence of gadolinium in solution: it can exist in the form of a molecular or colloidal form.

The probable origin of molecular gadolinium (Gd^3+^), which exists in the form of aquacomplexes [Gd(H_2_O)_7_]^3+^ [5], consists in contamination of the extraction product with gadolinium precursors during the synthesis of EMF (Gd(NO_3_)_3_, Gd_2_O_3_, etc.). The destruction of the carbon skeleton of Gd@C_82_ and the release of molecular gadolinium (Gd^3+^) from it due to ultrasonic treatment are unlikely due to the disparate energies of ultrasonic irradiation and the energy constants of the C–C bond break (605.0 ± 0.3 kJ/mol) [6]. The maximum energy parameters of the ultrasonic bath or horn used were 900 W of electrical power, which is approximately equivalent to 0.9 kJ/s, and the efficiency of converting electrical power to ultrasonic power is less than 100%. We have proposed options for the search for gadolinium forms in solution, based on the preliminary centrifugation of AFDs EMF followed by ICP–OES determination.

Colloidal gadolinium in form Gd@C_80_, Gd@C_82_ should be sedimented by centrifugation. Found that when the full visual sedimentation after 6 h, the solution was 6.2 % (wt.) content of gadolinium from the initial concentrations before centrifugation (table S6). A single-particle ICP-MS [7] should be used to check remains form of not-centrifuge Gd content.

| Table S6. Determination of Gd@C_82_ by means of ICP–OES at different time of centrifugation of AFDs Gd@C_82_ at 15000 rpm and the percentage of the deposited colloidal form of gadolinium (*n*=3, *P*=0.95) | | |
| --- | --- | --- |
| **Time of centrifugation, h** | ***с*_Gd_, ppm** | **Fraction of** **Gd@C_82_ from initial content, %** |
| 0 | 1.30 ± 0.10 | 100 |
| 2 | 0.89 ± 0.07 | 68.5 |
| 4 | 0.36 ± 0.05 | 27.7 |
| 6 | 0.08 ± 0.01 | 6.2 |

**REFERENCES**

[1] I.V. Kuvychko, A.V. Streletskii, A.A. Popov, S.G. Kotsiris, T. Drewello, S.H. Strauss, O.V. Boltalina, Seven-Minute Synthesis of Pure Cs-C60Cl6 from [60]Fullerene and Iodine Monochloride: First IR, Raman, and Mass Spectra of 99 mol % C60Cl6, Chemistry – A European Journal 11(18) (2005) 5426-5436.

[2] I.V. Kuvychko, A.A. Popov, A.V. Streletskii, L.C. Nye, T. Drewello, S.H. Strauss, O.V. Boltalina, Dynamic HPLC study of C70 chlorination reveals a surprisingly selective synthesis of C70Cl8, Chemical Communications 46(43) (2010) 8204-8206.

[3] R.F. Enes, A.C. Tomé, J.A.S. Cavaleiro, R. Amorati, M.G. Fumo, G.F. Pedulli, L. Valgimigli, Synthesis and Antioxidant Activity of [60]Fullerene–BHT Conjugates, Chemistry – A European Journal 12(17) (2006) 4646-4653.

[4] M. Maggini, G. Scorrano, M. Prato, Addition of azomethine ylides to C60: synthesis, characterization, and functionalization of fullerene pyrrolidines, Journal of the American Chemical Society 115(21) (1993) 9798-9799.

[5] D. Shriver, M. Weller, T. Overton, F. Armstrong, J. Rourke, Inorganic Chemistry, W. H. Freeman2014.

[6] W.M. Haynes, CRC Handbook of Chemistry and Physics, 96th Edition, CRC Press2015.

[7] D. Mozhayeva, C. Engelhard, A critical review of single particle inductively coupled plasma mass spectrometry – A step towards an ideal method for nanomaterial characterization, Journal of Analytical Atomic Spectrometry 35(9) (2020) 1740-1783.
